# Comparison between radical surgery and chemoradiotherapy in patients with cervical esophageal cancer: a propensity score matched analysis

**DOI:** 10.1186/s12893-023-02029-z

**Published:** 2023-05-11

**Authors:** Kang Qi, Gang Lin, Haibo Liu, Xining Zhang, Zhimao Chen, Jian Li

**Affiliations:** Department of Thoracic surgery, Peking University First Hospital, Xishiku str.8, 100034 Xicheng District, Beijing, China

**Keywords:** Cervical esophageal cancer, Chemoradiotherapy, Esophagectomy, Prognosis, SEER database

## Abstract

**Background:**

The prognostic value of radical surgery (RS) and chemoradiotherapy (CRT) for cervical esophageal cancer (CEC) was estimated using the Surveillance, Epidemiology and End Results (SEER) database after 1:1 propensity score matching (PSM).

**Methods:**

This retrospective study used SEER data of CEC patients between 2004 and 2015. The prognostic effects on cancer-specific survival (CSS) were evaluated using multivariate cox regression analysis following radical surgery or CRT before and after PSM. The subgroup analysis of CSS is carried out according to T stages.

**Results:**

A total of 440 patients met the eligibility criteria. Three hundred and fifty-six(80.9%)patients underwent chemoradiotherapy, and eighty-four (19.1%) patients underwent radical surgery. There were significant differences between patients of radical surgery and CRT groups with regard to the tumor grade, histology and N stage. After PSM, 80 matched pairs (A total of 160 patients) were selected. Multivariable cox regression analysis revealed no difference in the CSS of patients that underwent either radical surgery or CRT before [hazard ratio (HR): 0.955, 95% CI: 0.704–1.295, P = 0.766] and after PSM (HR: 0.767, 95% CI: 0.512–1.149, P = 0.198). Subgroup analysis revealed no significant difference in CSS between patients with radical surgery and CRT groups for all T stages (T 1–4, all P > 0.05).

**Conclusions:**

This analysis revealed that the prognostic outcomes in patients with cervical esophageal cancer were comparable between radical surgery and CRT.

## Background

The 5-year relative survival rate for esophageal cancer during 2009 through 2015 was 20%, the lowest for all cancers [1]. Cervical esophageal cancer (CEC), which extends from the level of the cricopharyngeal muscle to the thoracic inlet, accounting for less than 5% of all esophageal carcinomas [2]. The histopathological type of CEC is mainly squamous cell carcinoma (SCC)and local progress often occurs at the time of diagnosis [3]. Whether radical surgery (RS) or chemoradiotherapy (CRT) as standard treatments for cervical esophageal cancer are optimal is still controversial. Management of CEC differs from other segment of esophageal cancer because of the complicated structures around the cervical esophagus. Many patients undergoing cervical esophagectomy even require total pharyngolaryngectomy, which often results in various postoperative complications and compromise quality of life. Previous studies have shown that the 5-year OS(Overall Survival)rate of patients with cervical esophagectomy is only 12–27% [4, 5]. Therefore, chemoradiotherapy is often used as the standard treatment for patients with cervical esophageal cancer by European Society for Medical Oncology guidelines [6]. At present, the prognosis between chemoradiotherapy and surgery for patients with cervical esophageal cancer is still uncertain. The purpose of this study was to evaluate the treatment outcomes of radical surgery versus chemoradiotherapy in patients with cervical esophageal cancer.

## Methods

### Study Population

The data used in the current study was selected from the SEER(The surveillance, epidemiology, and end results)database using SEER*STAT 8.3.6 software. Patients with cervical esophagus cancer were identified using the International Classification of Diseases for Oncology (ICD-O) topography code of C15.0. The years of diagnosis were set to 2004–2015. The following information was extracted: age, gender, race, histological types, tumor stage and grade, chemoradiotherapy and radical surgery (esophagectomy and lymph node dissection), survival status and survival time. Patient inclusion criteria include: (I) an age ≥ 18 years; (II) A history of only one primary cervical esophagus cancer, according to the guidelines of the American Joint Committee on Cancer (AJCC, 6th Edition); (III)The treatment options include chemoradiotherapy or esophagectomy/radical surgery. Exclusion criteria include: (I) Patients with incomplete data and/or those that were lost to follow-up; (II) Only radiotherapy or chemotherapy in non-surgical cases; (III) In surgical cases, biopsy, radiofrequency ablation and endoscopic submucosal dissection; (IV) Cases with distant metastases were excluded.

### Statistical analyses

The chi-square test was used to evaluate the association between the surgical procedures and other clinicopathological factors. Kaplan–Meier (K–M) survival curves were assessed by the log-rank test. The prognostic outcomes referred to cancer specific survival (CSS), which only reflected deaths caused by cervical esophagus cancer. To estimate the impact of chemoradiotherapy or radical surgery on the prognosis, univariate and multivariate cox regression analysis was carried out. Propensity scoring is a balancing technique whereby a numerical value is assigned for the probability of an intervention or treatment. In order to solve the problem of imbalances in the baseline characteristics, we conducted propensity-matched(PSM)analysis for more objective comparisons. For PSM, patients receiving chemoradiotherapy or esophagectomy/Radical surgery were matched 1:1 with a caliper set at 0.02. The matching algorithm was nearest neighbor matching, and the estimation algorithm was logistic regression. All statistical calculations and PSM were performed using SPSS 25.0 (SPSS, Chicago, IL, USA) and Graph- Pad Prism 8.0 (GraphPad Software, San Diego, CA, USA). Two-tailed P values < 0.05 were considered statistically significant.

## Results

### Baseline characteristics

**Fig. 1:**
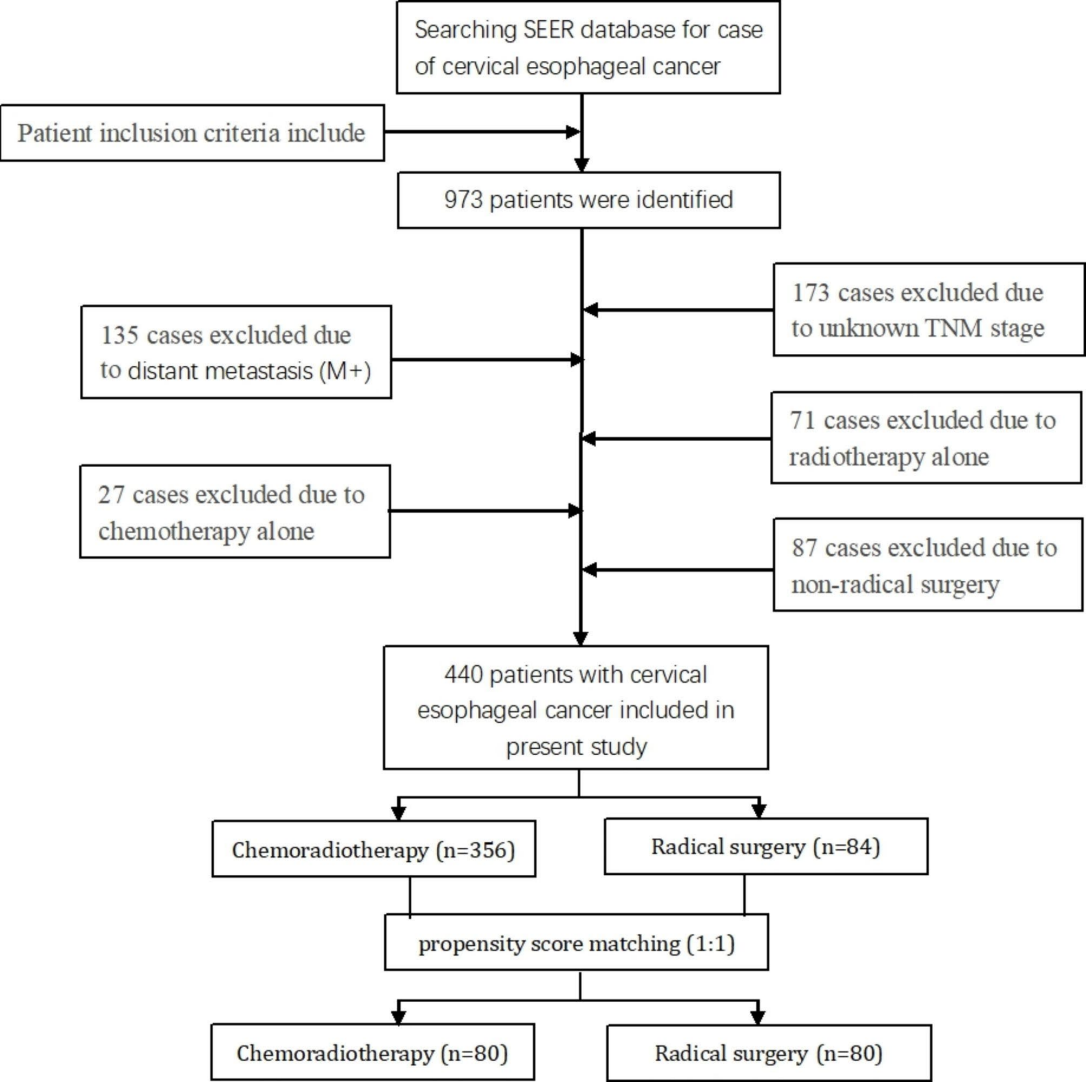
Flow-chart demonstrating the approach used to identify patients with cervical esophageal cancer registered in the SEER database from 2004 to 2015

A total of 973 patients with cervical esophageal cancer diagnosed between 2004 and 2015 from the SEER database. Among them, 440 patients meet the eligibility criteria and were included. Figure 1 illustrates the method used for data filtering the cervical esophageal cancer cases registered in the SEER database. Table 1 describes the demographic characteristics of the selected patients. There were 356 patients underwent chemoradiotherapy and 84 patients underwent radical surgery. In this patient population, 105 patients had cervical esophageal cancer staged at T1 stage, 43 patients had T2 stage,154 patients had T3 stage, and 140 had a T4 staging. Squamous cell carcinomas were more common than adenocarcinomas and other pathologic types (405 vs. 30 vs.5 patients).

**Table 1:** Characteristics of patients with CEC from the SEER database (n = 440)

Characteristic	No. of patients (%)
Year of diagnosis	
2004–2010	241(54.8)
2011–2015	199(45.2)
Age, years	
< 65	210(47.7)
≥ 65	230(52.3)
Sex	
Male	268(60.9)
Female	172(39.1)
Race	
White	341(77.5)
Black	66(15.0)
Other	33(7.5)
Tumor grade	
I	22(5.0)
II	205(46.6)
III	118(26.8)
Other	95(21.6)
Histology	
SCC	405(92.0)
AD	30(6.8)
Other	5(1.1)
T stage	
T 1	105(23.9)
T 2	43(9.8)
T 3	152(34.5)
T 4	140(31.8)
N stage	
N0	242(55.0)
N1	198(45.0)
Treatment	
RS	84(19.1)
CRT	356(80.9)

RS, Radical surgery; CRT, chemoradiotherapy; CEC, cervical esophageal cancer; SCC, Squamous cell carcinoma; AD, adenocarcinoma;

### Prognostic factors and CSS outcomes between patients with radical surgery or CRT before PSM

**Fig. 2:**
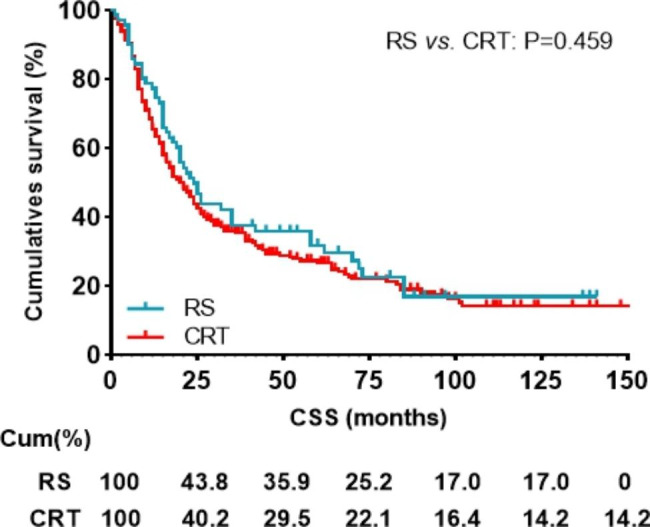
Impact of the radical surgery or CRT on CSS in patients with CEC before propensity score matching. CSS, cancer specific survival; RS, Radical surgery; CRT, chemoradiotherapy;

**Fig. 3:**
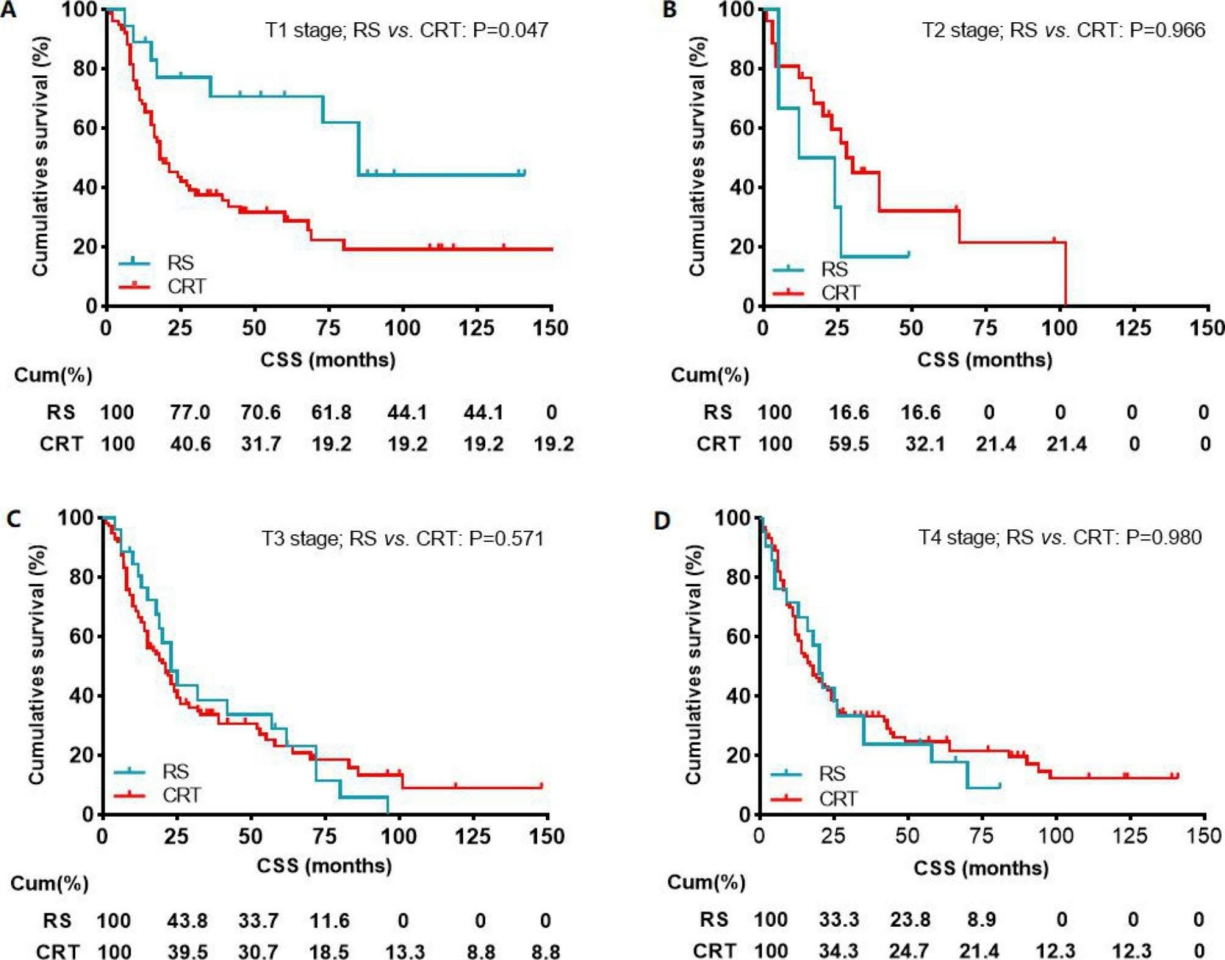
Impact of the radical surgery or CRT on CSS in patients with CEC stratified by T stage (**A**: T1 stage; **B**: T2 stage; **C**: T3 stage; **D**: T4 stage) before propensity score matching. CSS, cancer specific survival; RS, Radical surgery; CRT, chemoradiotherapy;

Before PSM, cox regression analysis of CSS showed that the prognostic outcome of patients with radical surgery or CRT was similar by univariate analysis (HR: 1.119, 95% CI: 0.831–1.505, P = 0.459) and multivariate analysis (HR: 0.955, 95% CI: 0.704–1.295, P = 0.766) (Table 2; Fig. 2). Univariate analysis showed that the prognostic factors affecting CSS were age (P = 0.018), sex(P = 0.004), histology(P = 0.009), and N stage(P = 0.003). Multivariate analysis showed that age (P = 0.023), sex (P = 0.001), histology (P < 0.006), and N stage(P = 0.003) were also independent prognostic factors for CSS (Table 2). Further subgroup analyses were made within the different T stages between patients with RS and CRT. Kaplan-Meier survival analysis and log-rank comparison revealed that the patients in T1 stage who receive RS had better CSS (P = 0.047) when compared with the patients receiving CRT (Fig. 3A). The CSS was not statistically different in patients with a T2 stage (P = 0.966), T3 stage (P = 0.517) and T4 stage (P = 0.980) (Fig. 3B-D).

**Table 2:** Univariate and multivariate analyses of the prognostic factors for CSS before PSM

Characteristic	Univariate			Multivariate		
	HR	95% CI	P value	HR	95% CI	P value
Year of diagnosis						
2004–2010	1.000					
2011–2015	1.021	0.798—1.305	0.871			
Age, years						
< 65	1.000			1.000		
≥ 65	1.328	1.049—1.681	0.018	1.318	1.039—1.672	0.023
Sex						
Male	1.000			1.000		
Female	0.694	0.543—0 0.887	0.004	0.651	0.508—0.836	0.001
Race						
White	1.000		0.446			
Black	1.202	0.875—1.653	0.256			
Other	0.901	0.564—1.441	0.663			
Tumor grade						
I	1.000		0.269			
II	1.631	0.923—2.883	0.092			
III	1.543	0.854—2.787	0.150			
Other	1.334	0.730—2.439	0.349			
Histology						
SCC	1.000		0.009	1.000		0.006
AD	0.453	0.264—0.777	0.004	0.426	0.245—0.749	0.002
Other	0.318	0.045—2.269	0.253	0.362	0.050—2.624	0.315
T stage						
T 1	1.000		0.169			
T 2	0.776	0.563—1.071	0.123			
T 3	0.742	0.466—1.181	0.208			
T 4	1.047	0.793—1.382	0.748			
N stage						
N0	1.000			1.000		
N1	1.419	1.122—1.793	0.003	1.378	1.091—1.761	0.008
Treatment						
RS	1.000			1.000		
CRT	1.119	0.831—1.505	0.459	0.955	0.704—1.295	0.766

RS, Radical surgery; CRT, chemoradiotherapy; CEC, cervical esophageal cancer; PSM, propensity score matching; SCC, Squamous cell carcinoma; AD, adenocarcinoma; HR, hazard risk; CI, confidence interval; CSS, cancer-specific survival; P < 0.05 represents significant difference

### Patient characteristics after PSM

**Fig. 4:**
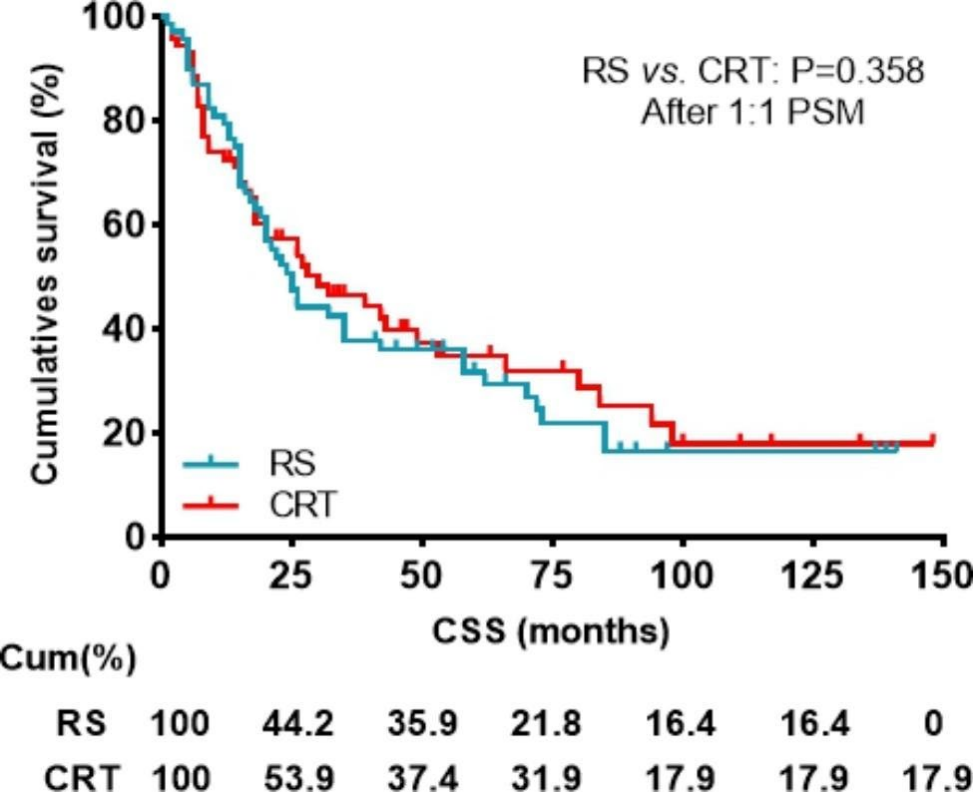
Impact of the radical surgery or CRT on CSS in patients with CEC after propensity score matching. CSS, cancer specific survival; RS, Radical surgery; CRT, chemoradiotherapy;

**Fig. 5:**
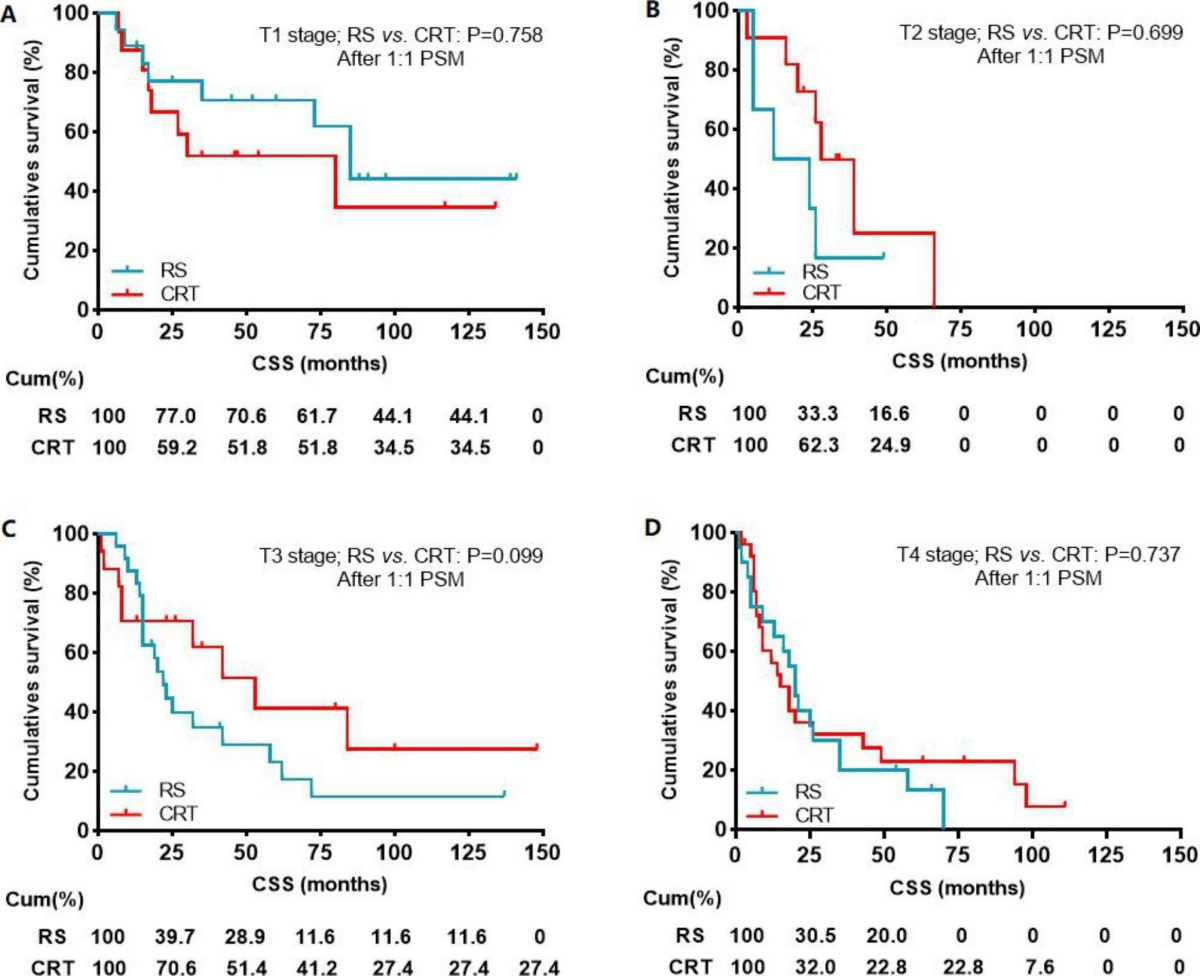
Impact of the radical surgery or CRT on CSS in patients with CEC stratified by T stage (**A**: T1 stage; **B**: T2 stage; **C**: T3 stage; **D**: T4 stage) after propensity score matching. CSS, cancer specific survival; RS, Radical surgery; CRT, chemoradiotherapy;

After 1:1 propensity score matching (PSM), baseline demographic and clinical variables were well balanced between patients that underwent RS and CRT (Table 3). Finally, 80 matched pairs (n = 160) were selected with balanced covariates. Univariate analysis (HR: 0.829, 95% CI: 0.556–1.236, P = 0.358) and multivariate analysis (HR: 0.767, 95% CI: 0.512–1.149, P = 0.198) for CSS showed that there was no difference in prognostic outcome of patients between RS and CRT after PSM (Table 4; Fig. 4). Subgroup analysis based on T stage was also carried out after PSM. Kaplan–Meier survival analysis and log-rank comparison revealed that there was no difference in CSS for all T stages (T1-4, all P > 0.05) after PSM (Fig. 5A-D).

**Table 3:** Characteristics of patients with CEC following RS or CRT before and after PSM

Characteristic	Before 1:1 PSM (%)				After 1: 1 PSM (%)		
	CRT(n = 356)	RS(n = 84)	P value		CRT(n = 80)	RS(n = 80)	P value
Year of diagnosis			0.033				0.870
2004–2010	187(52.5%)	54(64.3%)		49(61.3%)		51(63.7%)	
2011–2015	169(47.5%)	30(35.7%)		31(38.8%)		29(36.3%)	
Age, years			1.000				0.635
< 65	170(47.8%)	40(47.6%)		41(51.2%)		37(46.3%)	
≥ 65	186(52.2%)	44(52.4%)		39(48.8%)		43(53.8%)	
Sex			0.385				0.383
Male	213(59.8%)	55(65.5%)		60(75.0%)		54(67.5%)	
Female	143(40.2%)	29(34.5%)		20(25.0%)		26(32.5%)	
Race			0.163				0.791
White	271(76.1%)	70(83.3%)		65(81.3%)		68(85.0%)	
Black	59(16.6%)	7(8.3%)		8(10.0%)		7(8.8%)	
Other	26(7.3%)	7(8.3%)		7(8.8%)		5(6.3%)	
Tumor grade			0.060				0.646
I	15(4.2%)	7(8.3%)		4(5%)		6(7.5%)	
II	163(45.8%)	42(50%)		38(47.5%)		41(51.2%)	
III	93(26.1%)	25(29.8%)		30(37.5%)		23(28.7%)	
Other	85(23.9%)	10(11.9%)		8(10%)		10(12.5%)	
Histology			< 0.001				1.000
SCC	336(94.4%)	69(82.1%)		69(86.3%)		69(86.3%)	
AD	19(5.3%)	11(13.1%)		10(12.5%)		10(12.5%)	
Other	1(0.3%)	4(4.8%)		1(1.3%)		1(1.3%)	
T stage			0.902				0.349
T 1	86(24.2%)	19(22.6%)		19(23.8%)		19(23.8%)	
T 2	33(9.3%)	10(11.9%)		13(16.3%)		9(11.3%)	
T 3	123(34.6%)	29(34.5%)		18(22.5%)		27(33.8%)	
T 4	114(32.0%)	26(31.0%)		30(37.5%)		25(31.3%)	
N stage			< 0.001				1.000
N0	180(50.6%)	62(73.8%)		58(72.5%)		59(73.8%)	
N1	176(49.4%)	22(26.2%)		22(27.5%)		21(26.3%)	

RS, Radical surgery; CRT, chemoradiotherapy; CEC, cervical esophageal cancer; PSM, propensity score matching; SCC, Squamous cell carcinoma; AD, adenocarcinoma; P < 0.05 represents significant difference

**Table 4:** Univariate and multivariate analyses of the prognostic factors for CSS after PSM

Characteristic	Univariate			Multivariate		
	HR	95% CI	P value	HR	95% CI	P value
Year of diagnosis						
2004–2010	1.000					
2011–2015	1.190	0.754—1.879	0.454			
Age, years						
< 65	1.000					
≥ 65	1.185	0.794—1.768	0.407			
Sex						
Male	1.000					
Female	1.190	0.762—1.857	0.445			
Race						
White	1.000		0.173			
Black	1.829	0.971—3.444	0.061			
Other	1.036	0.478—2.244	0.929			
Tumor grade						
I	1.000		0.799			
II	1.296	0.585—2.874	0.523			
III	1.269	0.555—2.902	0.573			
Other	0.973	0.369—2.566	0.956			
Histology						
SCC	1.000		0.045	1.000		0.010
AD	0.396	0.113—0.847	0.013	0.396	0.191—0.821	0.013
Other	0.000	0.000—1.305	0.971	0.000	0.000—1.563	0.971
T stage						
T 1	1.000		0.057	1.000		0.125
T 2	1.663	0.787—3.512	0.183	1.608	0.756—3.413	0.217
T 3	1.901	1.042—3.468	0.036	1.686	0.915—3.108	0.094
T 4	2.253	1.272—3.990	0.005	2.035	1.137—3.640	0.017
N stage						
N0	1.000					
N1	1.155	0.735—1.814	0.531			
Treatment						
RS	1.000			1.000		
CRT	0.829	0.556—1.236	0.358	0.767	0.512—1.149	0.198

RS, Radical surgery; CRT, chemoradiotherapy; CEC, cervical esophageal cancer; PSM, propensity score matching; SCC, Squamous cell carcinoma; AD, adenocarcinoma; HR, hazard risk; CI, confidence interval; CSS, cancer-specific survival; P < 0.05 represents significant difference

oradiotherapy;

## Discussion

CEC is an uncommon disease and often locally advanced at time of diagnosis infiltrating many complicated anatomical structures adjacent to the cervical esophagus. Moreover, patients with CEC often have lymph node metastasis resulting in limited locoregional disease control and poor survival [7].

RS has been the standard treatment for CEC. Mostly, the surgical procedure includes the resection of the larynx and has a huge impact on quality of life [8]. The outcomes of patients undergoing radical CEC surgery have improved following the development of surgical techniques and strategies [9]. However, CEC surgery still leads to serious complications and high mortality [10, 11].

In recent years, noninvasive treatment such as CRT has been gradually explored and implemented. CRT treatment is often used for patients with unresectable tumors or for patients who cannot tolerate surgery. Recently, studies reported CRT has been used as the standard treatment for locally advanced cervical esophageal cancer [12]. Katsushi Takebayashi et al. reported RS and CRT as initial treatment for cervical esophageal cancer have comparable survival outcome [13]. Hoeben et al. reported locoregional recurrence rates in CEC patients treated with CRT(range from 13.7 to 42%) was slightly lower than that treated with radical surgery(range from 15.6 to 48.6%) [7]. Nevertheless, CRT may be accompanied by severe side effects and complications. Many patients are unable to tolerate the toxic and side effects of CRT, resulting in the failure of non-invasive treatment [14]. Michele Valmasoni et al. found that local recurrence was significantly higher in the CRT group than radical surgery (CRT 84% vs. SURG 50%) [12]. For non-complete response in patients with CRT, salvage surgery represents an acceptable treatment. 73.3% of patients with residual tumor after CRT could undergo salvage surgery. Salvage surgery can effectively complement CRT [13]. As can be seen from the above studies, the current standard for its treatment of CEC remains undetermined [15, 16]. In the United States and European countries, the treatment standard for CEC has been CRT [17]. However, centers in other countries may choose surgical treatment for CEC [5, 18].

This study aimed to compare the efficacy of RS and CRT in the treatment of cervical esophageal cancer. We found no difference in the prognostic outcome of patients that underwent either CRT or radical surgery, both by multivariate and univariate analysis. Further subgroup analyses revealed that the patients in T1 stage who receive radical surgery had better CSS compared with the patients receiving CRT. Because there were imbalances in the baseline characteristics between patients who underwent CRT and radical surgery, the variables affecting CSS were balanced after PSM. We found that CSS was also similar between CRT or RS after PSM. In addition, the results of our study showed no difference in the CSS between patients that underwent CRT or RS in all T stages.

There were several limitations in this study. The present study is a retrospective SEER analysis, so selection bias was inevitable. Postoperative complications and details of the chemotherapy and radiotherapy methods, including the sequence of treatment regiments, total dose and treatment techniques, were not provided by the SEER database. In our subgroup analysis, the survival time of patients with cervical esophageal cancer at T2 stage was significantly worse than that of patients with other stages. We analyzed that this was due to the deviation caused by the small number of patients enrolled in T2 stage, which does not reflect the true prognosis. To overcome this shift, more patients will need to be enrolled in future studies. On the other hand, in order to achieve a balance in the baseline characteristics between RS and CRT groups through PSM, four patients were lost in the RS group. We will include more patients in the RS group in the future to reduce the impact of data loss on the study. The treatment of cervical esophageal cancer remains a debated topic, additional prospective randomized trials are warranted to compare prognostic outcomes of RS and CRT.

## Conclusions

Using SEER data, we revealed that the prognostic outcomes in patients with cervical esophageal cancer were comparable between RS and CRT.

## Data Availability

Surveillance, Epidemiology, and End Results (SEER) belongs to public databases(https://seer.cancer.gov/). The patients involved in the database have obtained ethical approval. Users can download relevant data for free for research and publish relevant articles. Our research is based on open-source data, so there are no ethical issues and other conflicts of interest.

## Abbreviations

RS: Radical surgery
CRT: Chemoradiotherapy
CEC: Cervical esophageal cancer
SEER: Surveillance, Epidemiology and End Results
PSM: Propensity score matching
CSS: Cancer-specific survival
OS: Overall survival
CI: Confidence intervals
HR: Hazard ratios

## References

[1] Siegel RL, Miller KD, Jemal A (2020). Cancer statistics, 2020. CA Cancer J Clin.

[2] Lee DJ, Harris A, Gillette A, Munoz L, Kashima H (1984). Carcinoma of the cervical esophagus: diagnosis, management, and results. South Med J.

[3] Grass GD, Cooper SL, Armeson K, Garrett-Mayer E, Sharma A (2015). Cervical esophageal cancer: a population-based study. Head Neck.

[4] Mendenhall WM, Sombeck MD, Parsons JT, Kasper ME, Stringer SP, Vogel SB (1994). Management of cervical esophageal carcinoma. Semin Radiat Oncol.

[5] Daiko H, Hayashi R, Saikawa M, Sakuraba M, Yamazaki M, Miyazaki M (2007). Surgical management of carcinoma of the cervical esophagus. J Surg Oncol.

[6] Lordick F, Mariette C, Haustermans K, Obermannová R, Arnold D (2016). Oesophageal cancer: ESMO Clinical Practice Guidelines for diagnosis, treatment and follow-up. Ann Oncol.

[7] Hoeben A, Polak J, Van De Voorde L, Hoebers F, Grabsch HI, de Vos-Geelen J (2016). Cervical esophageal cancer: a gap in cancer knowledge. Ann Oncol.

[8] Archibald S, Young JE, Thoma A (2005). Pharyngo-cervical esophageal reconstruction. Clin Plast Surg.

[9] Tepper J, Krasna MJ, Niedzwiecki D, Hollis D, Reed CE, Goldberg R (2008). Phase III trial of trimodality therapy with cisplatin, fluorouracil, radiotherapy, and surgery compared with surgery alone for esophageal cancer: CALGB 9781. J Clin Oncol.

[10] Shuangba H, Jingwu S, Yinfeng W, Yanming H, Qiuping L, Xianguang L (2011). Complication following gastric pull-up reconstruction for advanced hypopharyngeal or cervical esophageal carcinoma: a 20-year review in a chinese institute. Am J Otolaryngol.

[11] Kadota H, Sakuraba M, Kimata Y, Hayashi R, Ebihara S, Kato H (2009). Larynx-preserving esophagectomy and jejunal transfer for cervical esophageal carcinoma. Laryngoscope.

[12] Valmasoni M, Pierobon ES, Zanchettin G, Briscolini D, Moletta L, Ruol A (2018). Cervical esophageal Cancer treatment strategies: a Cohort Study appraising the debated role of surgery. Ann Surg Oncol.

[13] Takebayashi K, Tsubosa Y, Matsuda S, Kawamorita K, Niihara M, Tsushima T (2017). Comparison of curative surgery and definitive chemoradiotherapy as initial treatment for patients with cervical esophageal cancer. Dis Esophagus.

[14] Gkika E, Gauler T, Eberhardt W, Stahl M, Stuschke M, Pöttgen C (2014). Long-term results of definitive radiochemotherapy in locally advanced cancers of the cervical esophagus. Dis Esophagus.

[15] Cao CN, Luo JW, Gao L, Xu GZ, Yi JL, Huang XD (2014). Primary radiotherapy compared with primary surgery in cervical esophageal cancer. JAMA Otolaryngol Head Neck Surg.

[16] Chou SH, Li HP, Lee JY, Huang MF, Lee CH, Lee KW (2010). Radical resection or chemoradiotherapy for cervical esophageal cancer?. World J Surg.

[17] Ajani JA, D’Amico TA, Bentrem DJ, Chao J, Corvera C, Das P (2019). Esophageal and Esophagogastric Junction Cancers, Version 2.2019, NCCN Clinical Practice Guidelines in Oncology. J Natl Compr Canc Netw.

[18] Ott K, Lordick F, Molls M, Bartels H, Biemer E, Siewert JR (2009). Limited resection and free jejunal graft interposition for squamous cell carcinoma of the cervical oesophagus. Br J Surg.

